# Supplementary material for: Cytokine Response of Natural Killer Cells to Hepatitis B Virus Infection Depends on Monocyte Co-Stimulation
Source: Viruses. 2024 May 8;16(5):741. doi: 10.3390/v16050741 (PMC11125674; doi:10.3390/v16050741)
Supplement: Supplementary file 1 [file viruses-16-00741-s001.zip › viruses-2958290-supplementary.pptx]

## Slide 1
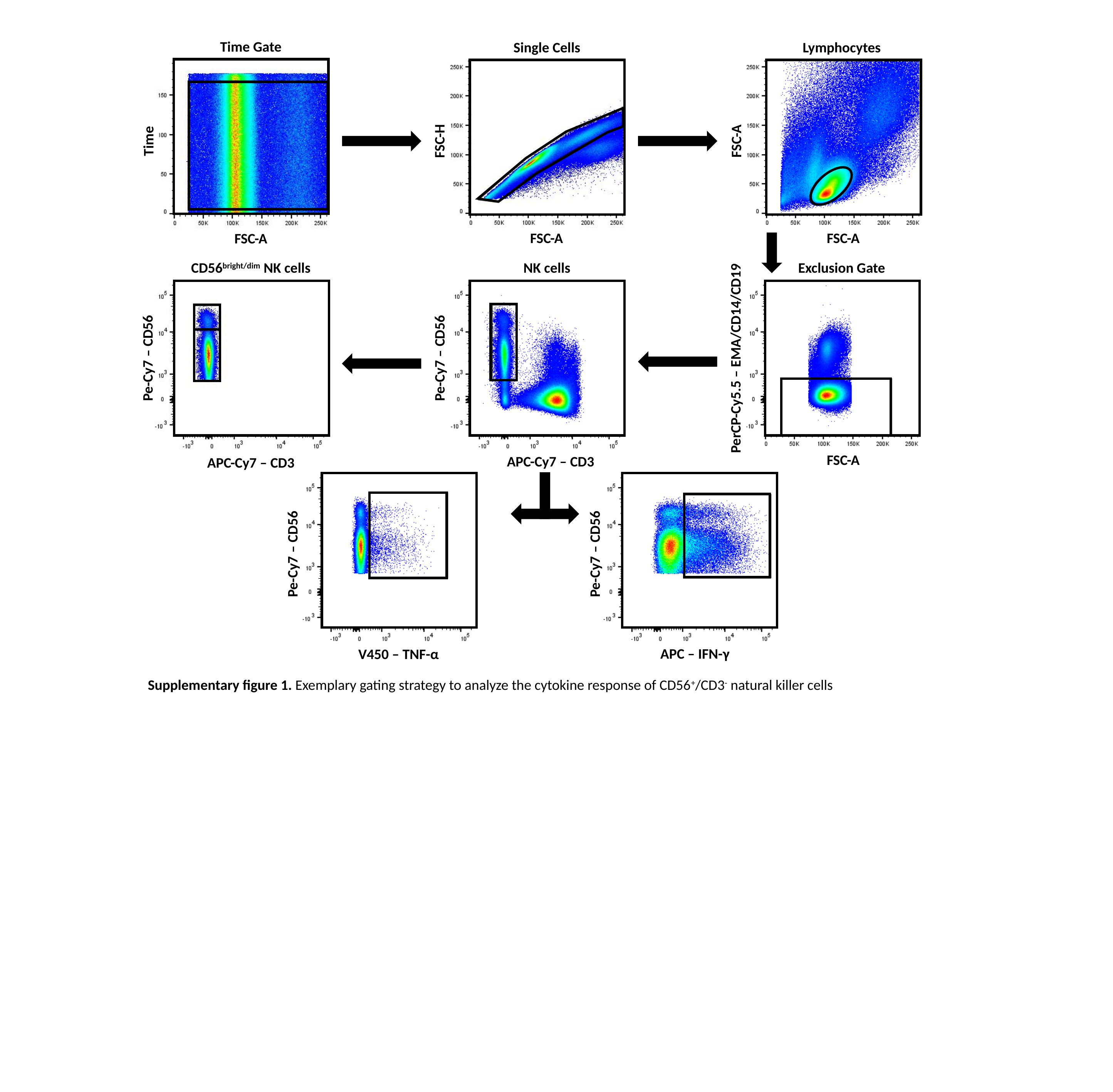

Time Gate
Single Cells
Lymphocytes
Time
FSC-H
FSC-A
FSC-A
FSC-A
FSC-A
CD56bright/dim NK cells
NK cells
Exclusion Gate
Pe-Cy7 – CD56
Pe-Cy7 – CD56
PerCP-Cy5.5 – EMA/CD14/CD19
FSC-A
APC-Cy7 – CD3
APC-Cy7 – CD3
Pe-Cy7 – CD56
Pe-Cy7 – CD56
APC – IFN-γ
V450 – TNF-α
Supplementary figure 1. Exemplary gating strategy to analyze the cytokine response of CD56+/CD3- natural killer cells

## Slide 2
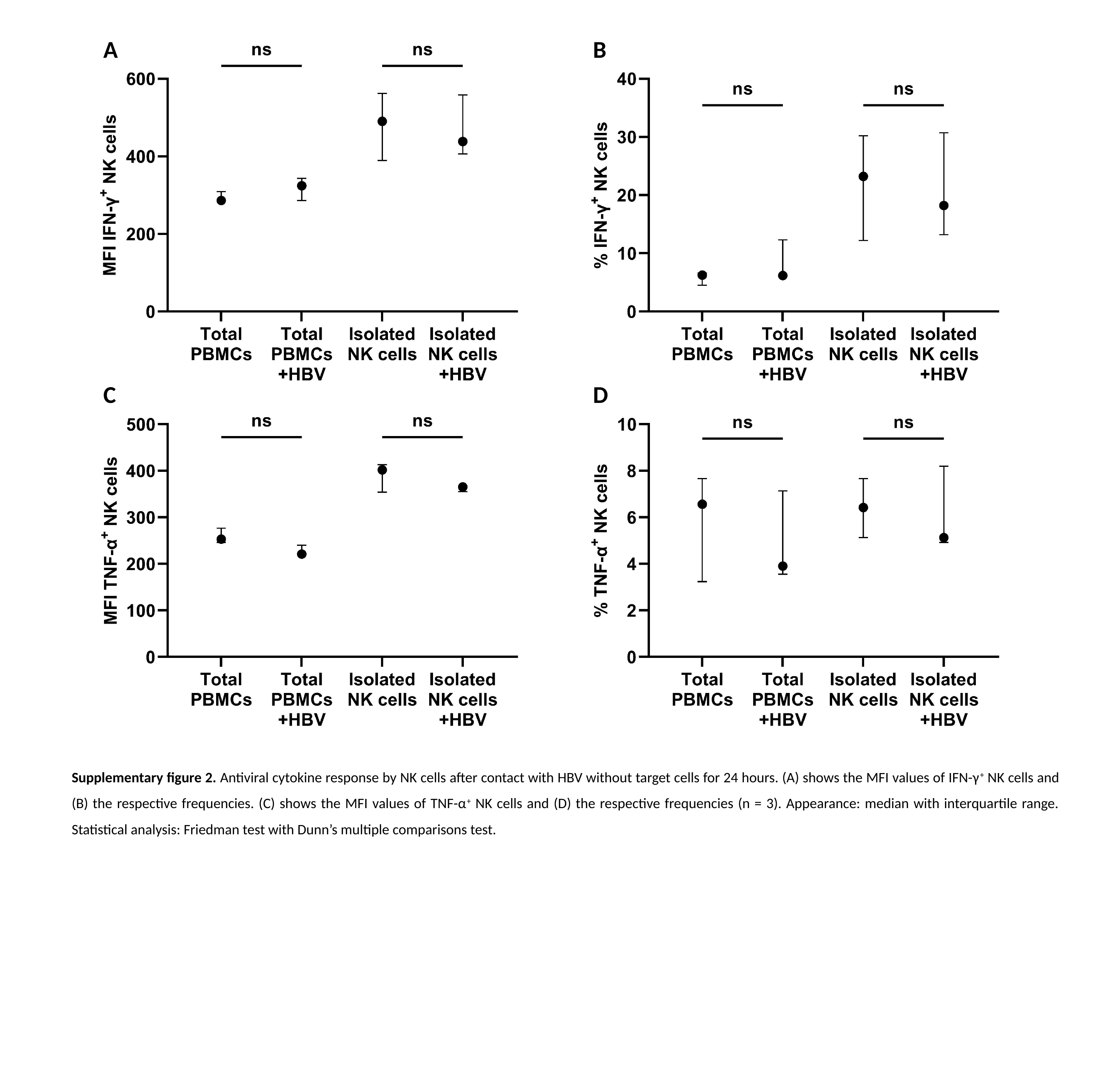

A
B
C
D
Supplementary figure 2. Antiviral cytokine response by NK cells after contact with HBV without target cells for 24 hours. (A) shows the MFI values of IFN-γ+ NK cells and (B) the respective frequencies. (C) shows the MFI values of TNF-α+ NK cells and (D) the respective frequencies (n = 3). Appearance: median with interquartile range. Statistical analysis: Friedman test with Dunn’s multiple comparisons test.
